# Effect of mitochondrial uncouplers niclosamide ethanolamine (NEN) and oxyclozanide on hepatic metastasis of colon cancer

**DOI:** 10.1038/s41419-017-0092-6

**Published:** 2018-02-13

**Authors:** Amer Alasadi, Michael Chen, G. V. T. Swapna, Hanlin Tao, Jingjing Guo, Juan Collantes, Noor Fadhil, Gaetano T. Montelione, Shengkan Jin

**Affiliations:** 1 Department of Pharmacology, Robert Wood Johnson Medical School, Rutgers - The State University of New Jersey, 675 Hoes Lane West, Piscataway, NJ 08854 USA; 2 Graduate Program of Physiology and Integrative Biology, Robert Wood Johnson Medical School, Rutgers - The State University of New Jersey, 675 Hoes Lane West, Piscataway, NJ 08854 USA; 3 Center for Advanced Biotechnology and Medicine, and Department of Molecular Biology and Biochemistry, Rutgers - The State University of New Jersey, 679 Hoes Lane West, Piscataway, NJ 08854 USA; 4 Clinical and Translational Science Program, Robert Wood Johnson Medical School, Rutgers - The State University of New Jersey, 675 Hoes Lane West, Piscataway, NJ 08854 USA; 5 Department of Biochemistry and Molecular Biology, Robert Wood Johnson Medical School, Rutgers - The State University of New Jersey, 675 Hoes Lane West, Piscataway, NJ 08854 USA

## Abstract

Metabolism of cancer cells is characterized by aerobic glycolysis, or the Warburg effect. Aerobic glycolysis reduces pyruvate flux into mitochondria, preventing a complete oxidation of glucose and shunting glucose to anabolic pathways essential for cell proliferation. Here we tested a new strategy, mitochondrial uncoupling, for its potential of antagonizing the anabolic effect of aerobic glycolysis and for its potential anticancer activities. Mitochondrial uncoupling is a process that facilitates proton influx across the mitochondrial inner membrane without generating ATP, stimulating a futile cycle of acetyl- CoA oxidation. We tested two safe mitochondrial uncouplers, NEN (niclosamide ethanolamine) and oxyclozanide, on their metabolic effects and anti-cancer activities. We used metabolomic NMR to examine the effect of mitochondrial uncoupling on glucose metabolism in colon cancer MC38 cells. We further tested the anti-cancer effect of NEN and oxyclozanide in cultured cell models, APC^min/+^ mouse model, and a metastatic colon cancer mouse model. Using a metabolomic NMR approach, we demonstrated that mitochondrial uncoupling promotes pyruvate influx to mitochondria and reduces various anabolic pathway activities. Moreover, mitochondrial uncoupling inhibits cell proliferation and reduces clonogenicity of cultured colon cancer cells. Furthermore, oral treatment with mitochondrial uncouplers reduces intestinal polyp formation in APC^min/+^ mice, and diminishes hepatic metastasis of colon cancer cells transplanted intrasplenically. Our data highlight a unique approach for targeting cancer cell metabolism for cancer prevention and treatment, identified two prototype compounds, and shed light on the anti-cancer mechanism of niclosamide.

## Introduction

During the first half of the 20th century, Otto Warburg observed that cancer cells metabolize glucose in a distinct manner^1,2^. Cancer cells tend to “ferment” glucose into lactate even in the presence of sufficient oxygen. This phenomenon of aerobic glycolysis is called “the Warburg effect”. Recent work in cancer cell metabolism has led to the elucidation of the significance of the Warburg effect to cancer^3,4^. In essence, glycolysis in non-dividing cells is followed by complete oxidation of pyruvate in mitochondria, producing the end product of CO_2_. This leads to a complete oxidation of glucose without biomass accumulation. In contrast, in cancer cells, the pyruvate flux into mitochondria is reduced as the result of aerobic glycolysis. It is estimated that pyruvate entering mitochondria for complete oxidation only represents ~5% of glucose metabolism, while a majority of pyruvate undergoes “fermentation” to lactate, which represents 85% of glucose metabolism. The remaining ~10% of glucose metabolism is shunted to other metabolic pathways, such as the pentose phosphate pathway (PPP). These pathways generate NADPH and metabolic intermediates such as ribose, providing the reducing agent and building blocks for biosynthesis required for biomass accumulation needed for cell proliferation^3–5^. Thus, targeting aerobic glycolysis, which in turn diminishes the production of reducing agents and building blocks for cancer cell biosynthesis, can be an effective and likely a universal anti-cancer strategy.

**Fig. 1:**
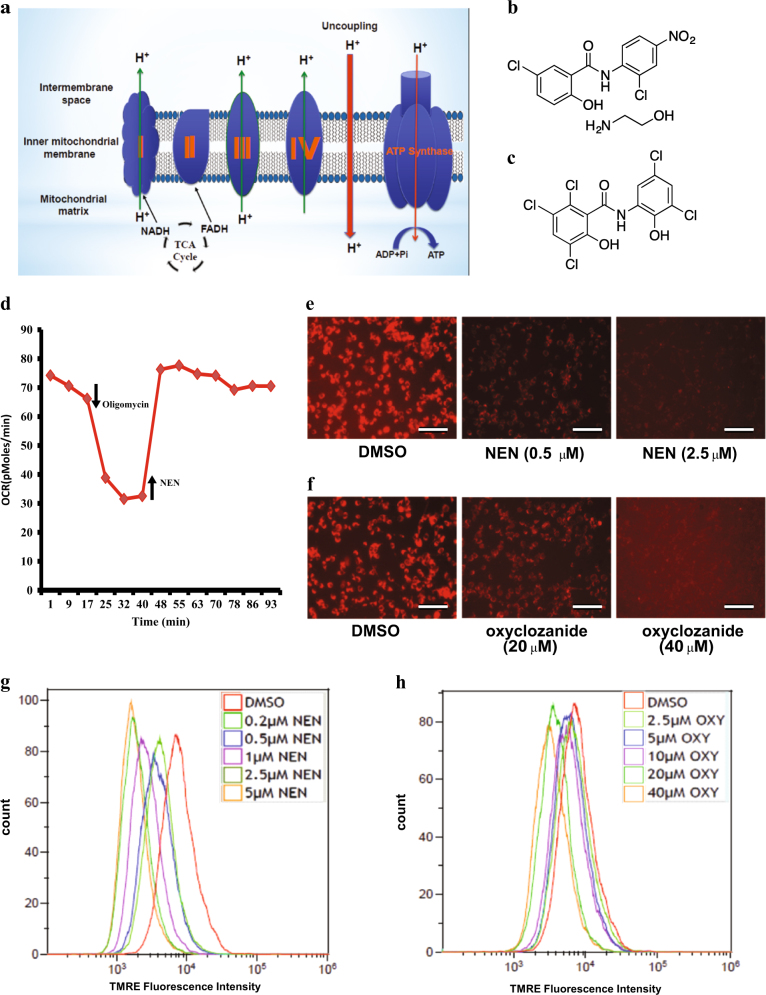
NEN and oxyclozanide uncouple mitochondria in cultured cells **a** Schematic representation showing mitochondrial uncoupling process. **b** Chemical structure of NEN. **c** Chemical structure of oxyclozanide. **d** Oxygen Consumption Rate (OCR) of cultured cells with sequential addition of oligomycin (final concentration 2.5 μM) and NEN (final concentration 2.0 μM), as indicated. **e, f** determination of minimal efficacious concentrations of NEN **e** and oxyclozanide **f** for mitochondrial uncoupling in murine colon cancer MC38 cells, scale bars, 200 μm. MC38 cells were treated with various concentrations of NEN or oxyclozanide while the control group was treated with vehicle DMSO for 2 h, followed staining with (TMRE) for 10 min. **g, h** quantification of TMRE staining by flow cytometry analyses. Results shown are representative data from three independent experiments.

Inside mitochondria, as illustrated in Fig. 1a, acetyl-CoA is metabolized to CO_2_ through TCA cycle, and energy is extracted and stored in the form of high-energy electrons in NADH and FADH_2_. The electrons then feed into the electron transport chain (ETC) residing in the mitochondrial inner membrane, which pumps protons out across the membrane and generates a proton gradient. Protons enter the mitochondrial matrix through ATP synthase, driving ATP synthesis. Usually, the ETC activity is coupled to the energy requirement of the cells. When the energetic requirement is met, ETC and oxidation of acetyl-CoA are shut down, along with pyruvate flux into mitochondria.

Mitochondrial uncoupling is a process that leads to proton influx across the mitochondrial inner membrane without passing through ATP synthase^6–9^. As illustrated in Fig. 1a, this process de-couples mitochondrial oxidation from ATP synthesis, leading to a futile cycle, i.e., complete oxidation of acetyl-CoA without generating ATP^6–10^. As a result, the energy efficiency of mitochondria is compromised. To meet the cellular energy demand, the flux of pyruvate into mitochondria is expected to accelerate, which promotes the complete oxidation of glucose. This mode of metabolic change induced by mitochondrial uncoupling could potentially diminish the anabolic effect of aerobic glycolysis.

Niclosamide was an FDA approved anthelmintic drug for treating tapeworm infection^11,12^. Its mechanism of action is to uncouple mitochondria^11–13^. Recent studies have found that niclosamide has strong in vitro anti-cancer activity against a wide arrange of cancer cells, including cells from colon cancer^14,15^, breast cancer^16–20^, glioma^21^, hepatocellular carcinoma^22^, adrenocortical carcinoma^23^, ovarian tumor^24^, osteosarcoma^25^, prostate cancer^26,27^, and many other cancer types^28,29^. The direct target of niclosamide in these studies was not identified and many potential anti-cancer pathways/mechanisms have been proposed, including Wnt/β-catenin^18,20^, Stat3^20^, NF-κB^30^, S100A4^14^, CDC37 Signaling pathway^22^, among others^22,28^. Oxyclozanide is another mitochondrial uncoupling anthelmintic drug approved for veterinary use^31^. Oxyclozanide has not yet been reported in literature to have any anti-cancer activity. We decided to use niclosamide and oxyclozanide as prototype drugs to examine the impact of mitochondrial uncoupling on cancer glucose metabolism and to determine their potential anti-cancer activities and particularly in vivo anticancer efficacy.

NEN (niclosamide ethanolamine) is the ethanolamine salt of niclosamide, which has a similar excellent safety profile as niclosamide^11,12,32,33^ and better systemic exposure^33^. Our recent studies showed that NEN has high levels of distribution in the liver after oral administration, where it uncouples mitochondria, reduces liver steatosis, and increase insulin sensitivity^34^. Similarly, oxyclozanide is also enriched in liver after oral administration^31,35^. Thus, we choose liver as a therapeutic site for NEN and oxyclozanide. Liver is one of the most important target organs for metastatic cancers, in particular for colorectal cancer. As a proof-of-concept, we used colorectal cancer model systems in our present studies to determine the effect of mitochondrial uncoupling on cell metabolism, tumorigenesis, and hepatic metastasis.

## Results

### Mitochondrial uncoupling by NEN and oxyclozanide

The hallmark of mitochondrial uncoupling is to induce mitochondrial oxygen consumption in the presence of a mitochondrial ATP synthase inhibitor such as oligomycin (Fig. 1a). The chemical structures of NEN and oxyclozanide are shown in (Figs. 1b and c) respectively. Using Seahorse oxygen consumption rate (OCR) assay, we confirmed that NEN uncouples mitochondria at 2.0 μM (Fig. 1d). Similar results were obtained with oxyclozanide at minimal concentration of 20 μM (data not shown). Mitochondrial uncoupling is often associated with a reduction of mitochondrial membrane potential, which serves a more robust method to accurately determine the minimal concentration required for inducing mitochondrial uncoupling. Previously, our group demonstrated that NEN uncouples mitochondria and reduces mitochondrial membrane potential in cultured fibroblast cells starting at 0.5 μM^34^. Here we demonstrated that NEN is efficacious in uncoupling mitochondria in the murine colon adenocarcinoma MC38 cells starting at the concentration 0.5 μM (Figs. 1e, g). The minimal mitochondrial uncoupling concentration of oxyclozanide is 20 μM (Figs. 1f, h). Similar results were obtained with other colon cancer cells, including human colon cancer HCT116 cells (Supplementary Fig. S1). To rule out the possibility that these compounds may exert their effects on the plasma membrane and thereby indirectly affect mitochondrial membrane potential, we tested the effect of NEN and oxyclozanide on the plasma membrane potential in the MC38 using DiBAC4(3) dye. Our result indicated that at the lower concentration spectrum under which NEN and oxyclozanide could reduce mitochondrial membrane potential, they had no clear effect on the plasma membrane potential (Supplementary Fig. S2), indicating that the effect of NEN and oxyclozanide on mitochondrial membrane potential is not a consequence of change of plasma membrane potential. At much higher concentrations NEN or oxyclozanide did cause a decrease of plasma membrane potential of MC38 cells, which is likely due to a sustained reduction of cellular ATP concentration as a result of extensive mitochondrial uncoupling (Supplementary Figure S2). Together, these studies confirmed that NEN and oxyclozanide are mitochondrial uncouplers and determined the efficacious concentrations for uncoupling in colon cancer cells such as MC38 and HCT116 cells.

### Mitochondrial uncoupling by NEN increases pyruvate flux into mitochondria and attenuates PPP

**Fig. 2:**
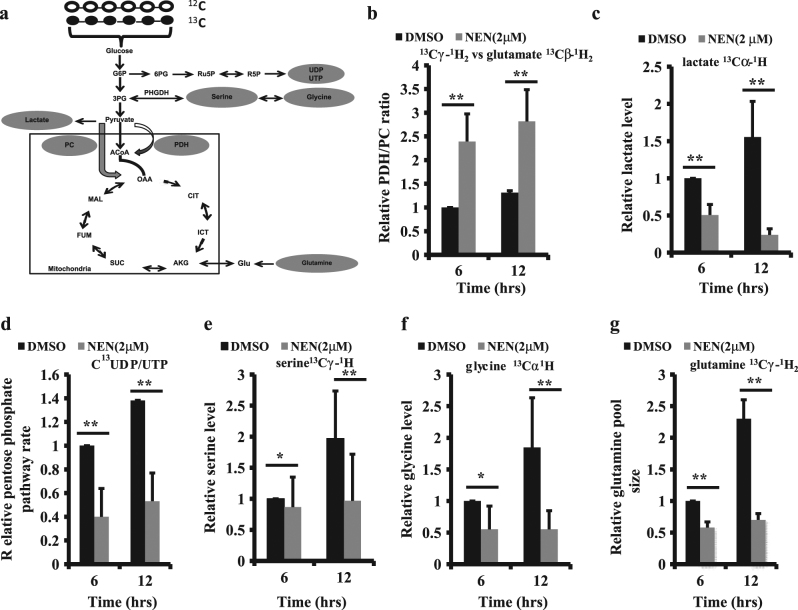
NEN inhibits the anabolic effect of aerobic glycolysis on colon cancer cell **a** Schematic of pathways and molecules measured in metabolomic NMR experiment. **b** Relative PDH/PC ratio determined by the ratio of glutamate^13^Cγ-^1^H_2_ vs. glutamate^13^Cβ− ^1^H_2_. **c** Relative lactate level determined by measuring lactate^13^Cα−^1^H_3_. **d** Pentose phosphate pathway (PPP) rate determined by measuring UDP/UTP^13^C labeling at ribose C2 position. **e** Relative serine level determined by measuring serine^13^Cγ-^1^H_3_. **f** Relative glycine level determined by measuring glycine^13^Cα-^1^H_3_. **g** relative glutamine level determined by measuring^13^Cγ-^1^H_2_. For **b–g** MC38 cells were grown in medium containing 50%^12^C + 50% U-^13^C glucose, treating with 2 μM NEN or vehicle DMSO for 6 or 12 h as indicated. The cell metabolites were extracted using cold methanol-chloroform extraction process. Abbreviations: G6P, glucose-6-phosphate; 3PG, 3- phosphoglycerate; PHGDH, phosphoglycerate dehydrogenase; 6PG, 6- phosphogluconate; Ru5P, ribulose-5-phosphate; R5P, ribose-5- phosphate; ACoA, acetyl coenzyme A; GlcNAc, N-acetyl-glucosamine; OAA, oxaloacetate; ICT, isocitrate; AKG, a-ketoglutarate; SUC, succinate; FUM, fumarate; MAL, malate. PDH, pyruvate dehydrogenase; PC, pyruvate carboxylase; Glu, glutamine. Results are showed as means ± SD values from three independent experiments and statistical significance (*P*) was determined by student t*-* test: *P < 0.05; **P < 0.01; vs. DMSO control

We then chose MC38 cells to study the impact of NEN on glucose metabolism, in particular the pathways relevant to aerobic glycolysis as illustrated in (Fig. 2a), using a metabolomic NMR approach^36^. [U-^13^C]-enriched glucose was added to the culture medium of cells, and the metabolites of glucose were analyzed and compared under the conditions of metabolism with or without treatment with NEN at concentrations that induce mitochondrial uncoupling (Figs. 2b to g). The identification of each metabolite and representative 2D [^13^C-^1^H]-HSQC spectra are shown in Supplementary Table S1 and Fig. S3.

We assessed the pyruvate dehydrogenase (PDH) to pyruvate carboxylase (PC) rate by analyzing the ^13^C labeling pattern of glutamate at the Cγ position vs. the Cβ position (Fig. 2b)^37^. Our results revealed that the PDH/PC rate was dramatically increased in the NEN treated cells compared to the control cells, indicating that NEN treatment increases pyruvate flux into mitochondria for oxidization. Moreover, lactate (^13^Cα-^1^H) was also directly measured (Fig. 2c). Consistent with an increase in pyruvate influx to mitochondrial, cells treated with NEN exhibited lower lactate levels. The pentose phosphate pathway (PPP) activity was analyzed by measuring accumulation of UDP and UTP by monitoring the ^13^C labeling pattern of ribose C2 UTP or UDP^36,38^. Cells treated with NEN showed dramatically lower PPP activity (Fig. 2d). The PHGDH (phosphoglycerate dehydrogenase) pathway (one carbon pathway)^36^ shunt was also analyzed by measuring relative serine and glycine levels. Measuring serine^13^Cγ-^1^H_3_ as shown in (Fig. 2e), and relative glycine levels determined by measuring glycine^13^Cα-^1^H_2_ (Fig. 2f). Similar to PPP activity, serine and glycine levels also decreased in NEN treated cells (Figs. 2e, f). Finally, cellular glutamine levels were measured (Fig. 2g). Consistent with increase in mitochondrial oxidation, cells treated with NEN exhibited dramatically reduced glutamine concentration. Together, the results demonstrated that NEN at concentrations that uncouple mitochondria leads to increased flux of pyruvate into mitochondria and increase in mitochondrial oxidation, which are accompanied by lower PPP and PHDGH activity, and a diminished glutamine pool.

### NEN and oxyclozanide cause G_0_/G_1_ cell cycle arrest and reduce the clonogenicity of colon cancer cells

**Fig. 3:**
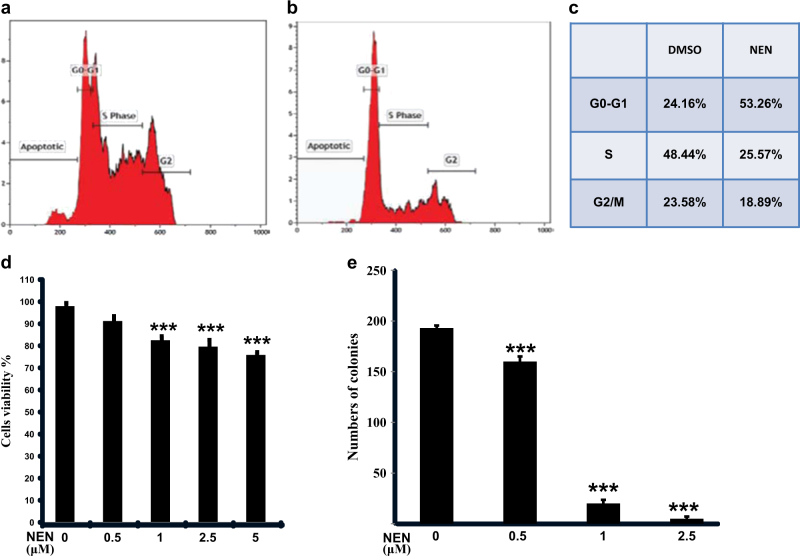
NEN affects cell cycle progression and reduces colony formation of colon cancer cells **a–c** Cell cycle profile of MC38 cells treated with DMSO vehicle (**a**) or 2.0 μM NEN (**b**) for 24 h, with percentage of cells in each phase summarized in **c**. **d** Cell viability of MC38 cells after a 24 h treatment with NEN at various concentrations, detected by trypan blue exclusion assay. **e** Clonogenicity of MC38 cells, cells were grown in medium containing NEN at various concentrations, as indicated, for 2 weeks, and the colonies formed were counted. Results from D-E are shown as means ± SD from three independent experiments and statistical significance (*P*) between the control and treated cells was determined by student *t*-test: ***P < 0.001

We next tested the effect of mitochondrial uncoupling on cell proliferation using NEN and oxyclozanide with the murine colon cancer cells MC38 and human colon cancer cells HCT116. NEN caused a significant accumulation of cells in G_0_/G_1_ with the concomitant decrease in cells in S phase compared to control group (Fig. 3a–c). Next, we sought to test NEN’s effect on cell viability. As shown in (Fig. 3d), a 24 h treatment with NEN at concentrations ranging from 0.5 to 5 μM led to reduced cell number by only 10~20%, likely due to slowing down cell growth and proliferation. The anti-cancer activity of NEN was further analyzed with clonogenic assay. In contrast to short term treatment, continuously exposure to low concentration of NEN led to over 90% (1 μM) to near 100% (2.5 μM) reduction in colony formation (Fig. 3e), indicating NEN has potent anti-proliferation effect in vitro. Similar results were obtained from HCT116 cells (Supplemental Fig. S4).

We then tested the uncoupling and anti-proliferation effect of oxyclozanide with MC38 and HCT116 cells lines. Oxyclozanide is a much less potent mitochondrial uncoupler and starts to uncouple mitochondria at concentrations of 20 μM (Fig. 1f). Similar to NEN, at concentrations efficacious for mitochondrial uncoupling, oxyclozanide increased cell accumulation in G_0_/G_1_ with the concomitant decrease in the number of cells in S phase compared to control cells (Supplemental Fig. S5a). Again, similar to NEN, while oxyclozanide has a minor impact on cell viability after short term treatment, at the concentrations that are active in mitochondrial uncoupling (20 μM) as shown in (Supplementary Figure S5b), it has a dramatic effect in reducing clonogenicity upon long term exposure at the same concentration (Supplementary Fig. S5c). The results support that like NEN, oxyclozanide has anti-proliferation activity.

### NEN and oxyclozanide inhibited cancer cells invasion and migration

We analyzed the effect of NEN and oxyclozanide on cell invasion and migration at concentrations that uncouple mitochondria. First, we tested the effect of NEN on colon cancer invasion using a Boyden chamber assay with MC38 cells. NEN treated cells showed a substantial inhibition in cell invasion when compared to the control group (DMSO vs. 1 μM NEN mean = 91 vs. 21%, *P* < 0.001) (Figs. S6a–c). Next, we analyzed the effect of NEN on colon cancer cell migration using a wound-healing assay. MC38 cells treated with vehicle completely closed the wound after 12 h treatment while NEN treated cells showed impaired migration, preventing the wound to close even after 12 h (Figs. S6 d–e). These results indicate that in addition to the effect on cell proliferation, short term NEN treatment could also inhibit colon cancer cell mobility, impairing invasion and migration. We repeated the cell migration experiments with oxyclozanide, which exhibited a similar inhibitory effect on cell migration at concentrations that uncouple mitochondria (Fig. S7).

### NEN reduced intestinal polyps in APC^min/+^ mice

**Fig. 4:**
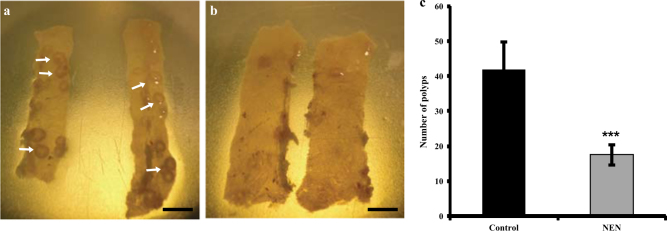
Oral NEN treatment reduces intestinal polyps in APC^min/+^ mice **a**, **b** Representative pictures of intestine fragments from control or NEN fed mice, respectively. Arrows point to areas containing polyps. Scale bars, 5 mm. **c** Quantification of intestinal polyps in control or NEN fed mice (*n* = 10 in each group). Mice at age of 2 months fed either normal chow diet or diet containing 1500 ppm NEN for 8 weeks. Polyps in intestine of each mouse were counted. Results shown as means ± SD. Statistical significance (*P*) was determined by student-*t* test between the control and NEN fed mice: ***P < 0.001. The data are representative results from two independent experiments

To test the potential in vivo anti-cancer effect on colon cancer, we treated the APC^min/+^ mice with NEN. The APC^min/+^ mice develop intestinal polyps at age of 4 months. At the age of 2 months, we fed mice with either normal diet or diet containing 1500 ppm NEN (equivalent to 120–150 mg/kg/day)^34^. After 8 weeks of treatment, mice were sacrificed and the intestinal polyps were analyzed. As shown in (Fig. 4a–c), oral NEN dramatically reduces polyp formation in this mouse model, supporting that NEN has anticancer activity against colon tumor in vivo.

### NEN and oxyclozanide reduced hepatic metastasis of colon cancer cells

**Fig. 5:**
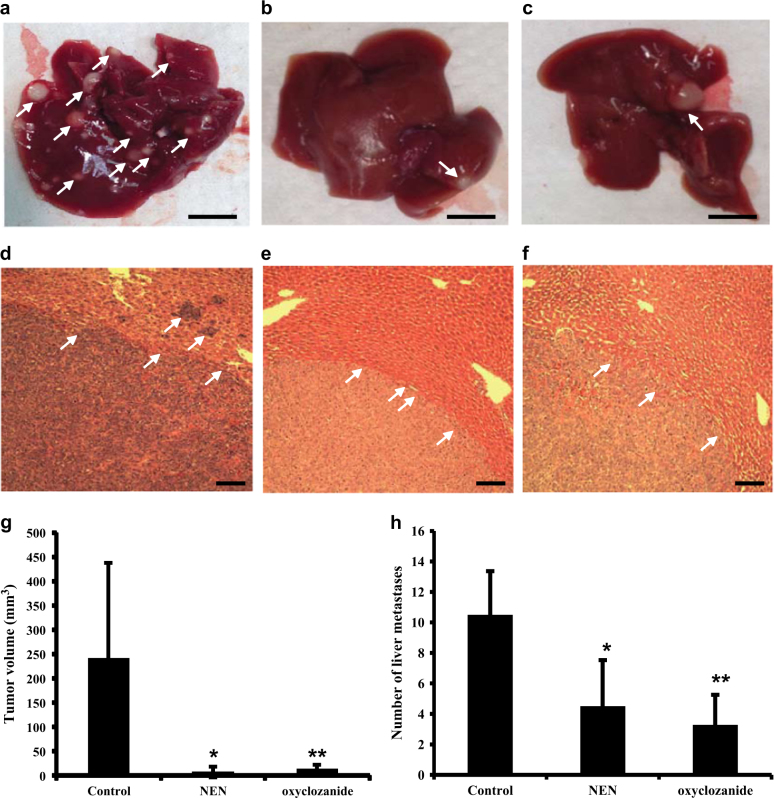
Effect of NEN and oxyclozanide on liver metastasis of colon cancer cells **a–c** Representative liver pictures (scale bars, 10 mm) showing the tumor nodules from mice fed normal chow (**a**) or diet containing 2000 ppm NEN, or diet containing 800 ppm oxyclozanide. Arrows point to area containing a tumor nodule. **d–f** Representative hematoxylin and eosin (H & E), (scale bars, 200 μm), staining of histological section of metastatic tumors mice fed normal diet (**d**), diet containing NEN (**e**), or diet containing oxyclozanide (**f**). Arrows point to boundary between tumous and normal tissues. **g–h**, average metastatic tumor volume (**g**) or number of node (h) per mouse in animals with indicated treatment. MC38 cells were injected into male NSG mice intrasplenically and randomized into 3 groups (*n* = 10 per group). The mice were fed normal chow, or chow containing 2000 ppm NEN, or chow containing 800 ppm oxyclozanide for 3 weeks, before euthanization and characterization of metastatic hepatic cancer. Numbers are presented by means ± SD. *P* value between control and each treated group was determined by student *t*-test: *P < 0.05 and **P < 0.01. The data show representative results from two independent experiments

Hepatic metastasis of colon cancer represents a clinical challenge. We examined the effect of NEN and oxyclozanide on colon cancer metastasis using a well-established mouse model^39^. The immune deficient NOD-scid gamma (NSG) mice were injected with MC38 cells intrasplenically. The colon cancer cells metastasize to liver if untreated (Figs. 5a, d). The mice were then randomized into three groups, one group fed normal diet, one group fed a diet containing 2000 ppm NEN, and the third group fed chow containing 800 ppm oxyclozanide. The concentration of NEN or oxyclozanide in food was determined by pharmacokinetic studies in literature^31,34^. Mice fed 2000 ppm NEN or 800 ppm oxyclozanide would give rise to plasma concentrations of NEN over the range from ~0.5 to ~3 μM^34^ or oxyclozanide ranging from ~20 to ~40 μM^31^, as oxyclozanide is much more stable metabolically in vivo than NEN. As shown in (Figs. 5b, c, e, f), NEN or oxyclozanide either completely prevented or drastically reduced hepatic metastasis of colon cancer cells from spleen (quantified in Figs. 5g, h).

### Mitochondrial uncoupling induced activation of AMPK and down regulated mTOR activity

**Fig. 6:**
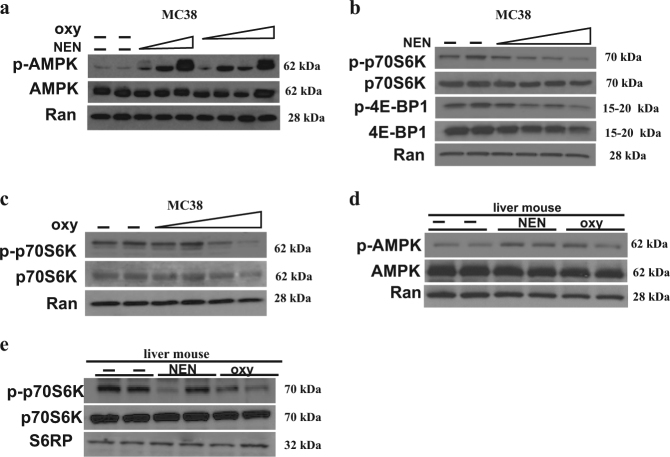
NEN and oxyclozanide activate AMPK and downregulate mTOR in vitro and in vivo **a–c** Immunoblot analyses of MC38 cells without or with treatment of NEN or oxyclozanide for 2 h with indicated antibodies. **d–e** Immunoblot analyses of mouse liver tissues from mice fed normal chow, or chow containing 2000 ppm NEN, or chow containing 800 ppm oxyclozanide for 3 weeks, with indicated antibodies. The data are representative results from two independent experiments

Using NMR metabolomics data, we directly showed that mitochondrial uncoupling by NEN changes glucose metabolism and antagonizes the anabolic effect of aerobic glycolysis, including diminished PPP pathway activity, increased pyruvate flux into mitochondria, and reduced glutamine pool. As mitochondria are also important bioenergetic organelles, we measured signal transduction pathways related to the bioenergetic changes. AMP-activated protein kinase (AMPK) is a master regulator of intracellular energy balance^40^. We treated MC38 cells with NEN or oxyclozanide and cells and analyzed AMPK activation. As shown in (Fig. 6a), NEN or oxyclozanide activated AMPK (increase in phosphorylated AMPK levels) in a dose dependent manner at concentrations that uncouple mitochondria.

Mammalian target of rapamycin (mTOR) is a conserved serine-threonine protein kinase, which regulates cellular growth and proliferation^41^. mTOR is one critical downstream target of AMPK (i.e., AMPK activation inhibits mTOR activity^42^). We tested if mitochondrial uncoupling leads to mTOR inhibition. Phosphorylation of p70SK and 4EBP1 can be used as readout for mTOR activity, which are mTOR’s direct substrates^43^. Fig. 6b showed that NEN downregulates mTOR activity (decreased phosphorylation of p70S6K and 4EBP1). Similar results were obtained with oxyclozanide treatment (Fig. 6c). Lastly, we tested whether mitochondria uncoupling could activate AMPK in vivo. Previous studies showed that both acute and chronic oral treatment activate AMPK in mouse liver^34^. Here we tested the effect of oxyclozanide on AMPK activation in mouse liver. Oxyclozanide was administered to mice via oral gavage and hepatic tissue was analyzed for AMPK activity 6 h later. As shown in (Fig. 6d), similar to NEN, oxyclozanide treatment leads to AMPK activation. In addition, our data demonstrated that the chronic oral treatment with NEN or oxyclozanide inhibited the mTOR activity in liver tissues as shown in (Fig. 6e).

## Discussion

Most, if not all, cancer cells exhibit an altered glucose metabolism mode, which is known as aerobic glycolysis or the Warburg effect^1,2,44^. This mode of metabolism prevents complete oxidation of glucose and shunts a substantial portion of glucose to biosynthetic pathways for biomass accumulation required for cell proliferation^5^. Targeting the unique glucose metabolism pathways in cancer cells has becoming an active field for therapeutic development. The critical regulatory step altered in aerobic glycolysis is the influx of pyruvate to mitochondria. While in differentiated cells almost all pyruvate molecules derived from glycolysis enter mitochondria for oxidative phosphorylation and the energy extraction from glucose is maximized; in cancer cells, it is estimated that only 5% of pyruvate enters mitochondria^5^. The aerobic glycolysis is accompanied by massive elevation of the rate of glucose uptake by cancer cells, which explains the lack of energetic deficiency in cancer cells despite the fact that only a small portion of glucose is used for oxidative phosphorylation. Aerobic glycolysis is functionally essential for cancer cell proliferation, as glucose is shunt to anabolic pathways such as PPP for production of NADPH and building blocks of macromolecules. Understanding the exact mechanisms leading to the reduction of pyruvate influx to mitochondria in various cancer cells and developing pharmacological intervention that facilitate the pyruvate influx are becoming important areas of cancer research.

In the present study, we tested whether mitochondrial uncoupling could alter the bioenergetics to promote pyruvate influx to mitochondria for complete oxidation and whether mitochondrial uncouplers have anti-cancer activity. Our metabolomics NMR results directly demonstrated that mitochondrial uncoupling by NEN dramatically increases pyruvate flux into mitochondria, increases mitochondrial oxidation, reduces lactate production, and reduces biosynthetic PPP pathway. Moreover, NEN and oxyclozanide exhibit potent anticancer activity both in inhibiting tumorigenesis in the APC^min/+^ mice and in preventing/reducing hepatic metastasis in an intrasplenic transplantation mouse model. These results support that mitochondrial uncoupling is an effective way of antagonizing aerobic glycolysis and safe mitochondrial uncouplers can be potentially developed for treating cancer. In addition to the effect on metabolism impacting biomass production essential for cell proliferation, mitochondrial uncoupling also has an impact on energy metabolism. The latter effect leads to AMPK activation, which in turn regulates cell cycle progression. It is likely that the bioenergetic effect of mitochondrial uncouplers also contributes to their anticancer activities.

One major mechanism by which pyruvate influx to mitochondria is regulated to meet the cellular bioenergetic demand is through a well-established negative feedback control involving PDH^45,46^. PDH catalyzes the conversion of pyruvate to acetyl-CoA, and PDH activity is inhibited by its end product, acetyl-CoA via the following mechanism: acetyl-CoA activates pyruvate dehydrogenase kinase, which phosphorylates and inactivates PDH^46^. Decrease in cellular ATP level, or more precisely, increase in cellular ADP level, activates mitochondrial oxidative phosphorylation, which accelerates mitochondrial TCA cycle and reduces mitochondrial acetyl-CoA concentration. In turn, PDH inhibition is relieved which promotes pyruvate influx to mitochondria. Mitochondrial uncoupling, which decouples mitochondrial ATP synthesis from electron transport chain activity, creates a futile cycle that could drastically increase mitochondrial oxidation of acetyl-CoA and decrease mitochondrial acetyl-CoA concentration, leading to PDH activation and pyruvate influx. This well-established metabolic negative feedback loop is likely the major mechanism underlying the observed results that mitochondrial uncoupling leads to increase in pyruvate influx into mitochondria in the cancer cells.

Niclosamide and oxyclozanide are well-documented mitochondrial uncouplers^11–13^. Niclosamide was an FDA approved anthelmintic drug for treating flatworm infection in gastrointestinal tract and mitochondrial uncoupling is the mechanism of action for this drug^11,12^. Our recent studies showed that oral NEN in mice leads to preferential distribution in liver^34^, resulting mitochondrial uncoupling that is effective for preventing and treating hepatic steatosis and insulin resistance. Importantly, NEN has excellent safety profiles in various mammalian species. Oxyclozanide is another mitochondrial uncoupling anthelmintic drug for veterinary use. Oxyclozanide is structurally related to niclosamide. It is less potent than niclosamide for mitochondrial uncoupling (minimal efficacious concentration of NEN is around 0.5 μM, while minimal efficacious concentration for oxyclozanide is 20–40 μM), but is metabolically much more stable in animals^31^. We showed here that not only NEN and oxyclozanide have potent anti-cancer activities both in vitro and in vivo, but also their  anticancer effects are associated with their mitochondrial uncoupling activity. Niclosamide has been shown to have anticancer activity in previous reports^14,20,47^, which attributed to the anti-cancer activity to its inhibitory effects on Wnt- or Stat3- pathways or on S100A4^14,20,47^. However, in vitro studies with NCI 60 human cancer cell lines indicate that niclosamide inhibits cell growth in all tested cancer cell lines^48^. The GI_50_ (growth inhibition) defined in this study is the same for all tumor cells (~500 nM), which is consistent with the efficacious mitochondrial uncoupling concentrations in mammalian cells documented in our published^34^ and current studies. The NCI-60 data thus support our hypothesis that the universal growth inhibitory effect and the anti-cancer activity of niclosamide are related to mitochondrial uncoupling rather than targeting a specific oncogenic pathway mutated in certain tumors. The observed changes in niclosamide treated cells in the above mentioned studies might be secondary to mitochondrial uncoupling which may also contribute to its anti-cancer activities in those specific cancer types.

In summary, we have shown that mitochondrial uncoupling changes cancer cell metabolism, which antagonizes the anabolic effect of aerobic glycolysis. Moreover, our data demonstrated that mitochondrial uncouplers NEN and oxyclozanide have potent anti-cancer activities for treating hepatic metastatic tumors in mouse model. These results support a new anti-cancer strategy for targeting the universal metabolic change in cancer, the Warburg effect. Our study also provided prototype compounds, which could be potentially derivatized based on the mitochondrial uncoupling activity for the development of new anti-cancer therapeutics.

## Materials and methods

### Cell lines

Murine colon adenocarcinoma cell line (MC38) was a kind gift from Dr. Harvey Roy Herschman (University of California Los Angeles, CA), Human colon carcinoma cell line HCT116 was a gift from Dr. Steven Zheng (Rutgers University of New Jersey), Mouse myoblast cells (C2C12) was purchased from the American Type Culture Collection (ATCC, Manassas, VA, USA). MC38, HCT116 and C2C12 cells were maintained in Dulbecco’s Modified Eagle Medium (DMEM) containing 10% FBS, 0.1 mM nonessential amino acids, 1.0 mM sodium pyruvate, and 1% penicillin-streptomycin, and incubated at 37 °C and 5% CO_2_.

### Reagents and antibodies

NEN (niclosamide 5-chloro-salicyl-(2-chloro-4-nitro) anilide 2-aminoethanol salt), was purchased from 2A PharmaChem (Lisle, IL). Oxyclozanide, trypan blue, crystal violet, glucose and DSS (4,4-dimethyl-4-silapentane-1-sulfonic acid), propidium iodide (PI), RNAse A, and tetramethylrhodamine ethyl ester (TMRE) were purchased from Sigma. DiBAC4(3) was purchased from (Invitrogen/ Thermofisher). [U-^13^C] glucose and 99.9% enriched deuterium oxide (D_2_O) were purchased from Cambridge Isotope Laboratories (Tewksbury, MA). Antibodies against pAMPK-(Thr172), AMPK, p-p70S6 kinase (Thr-389), p4E-BP1, 4E-BP1 and S6RP were purchased from Cell Signaling Technology (Danvers, MA). Ran antibody was from Santa Cruz Biotechnology (Dallas, TX).

### Oxygen consumption rate (OCR)

OCR analyses were performed using the Seahorse XF24 cartridge according to the instructions from Agilent technologies. Briefly, 40,000–50,000 mouse myoblast cells (C2C12) were seeded in a Seahorse 24XF cell culture microplate and cultured in DMEM medium. Before the analyses, cells were washed with DMEM media and placed in a non-CO_2_ 37 °C incubator for at least 1 h_._ OCR of the cells was analyzed by stepwise injections of 2.5 μM oligomycin and 2.5 μM NEN into each well.

### Mitochondrial membrane potential assay

Murine colon adenocarcinoma (MC38) or human colon carcinoma (HCT116) cells were plated onto six-well plates and maintained in DMEM until reaching 70% confluence. Then, cells were treated with various concentrations of NEN or oxyclozanide for 2 h, followed by staining with 100 nM TMRE for 10–15 min. Finally, cells were rinsed once with Dulbecco’s phosphate-buffered saline (DPBS) and then observed under fluorescence microscopy.

In order to quantify the TMRE staining after treatment with NEN and oxyclozanide, we performed flow cytometry analysis. MC38 cells were plated in 100 mm dish and maintained in DMEM until reaching 75% confluence. Then, cells were treated with various concentrations of NEN or oxyclozanide for 2 h, followed by staining with 100 nM (TMRE) for 15–30 min at 37 °C. After that, the cells were trypsinized and the cells pellet was collected by centrifuge (1000 rpm) for 5 min at room temperature. Finally, the cells pellet resuspended in 700 µl of DPBS and the TMRE fluorescence intensity and distribution was detected by flow cytometry.

### Measurement of plasma membrane potential

Plasma membrane potential of MC38 was measured using DiBAC4(3) fluorescence dye. MC38 cells were plated in 100 mm dish at concentration (2 × 10^6^ cells/ml) and maintained in DMEM. Then, the cells were treated with different concentration of NEN or oxyclozanide for 2 h, followed by staining with 5μM DiBAC4(3) for 30 min at 37 °C. Then, the cells pellet was collected and suspended in 500 μl DPBS and fluorescence intensity of DiBAC4(3) was detected by flow cytometry.

### Cell culture medium for NMR metabolomics experiments

Prior to the experiment, MC38 cells were maintained in DMEM containing 10% FBS, 0.1 mM nonessential amino acids, 1.0 mM sodium pyruvate, and 1% penicillin-streptomycin. For NMR labeling experiments, MC38 cells were seeded into 150 mm dish at a density 6 × 10^6^ cell per dish. After overnight incubation, the medium was replaced with labeled DMEM phenol—free medium containing 10% FBS, 1% penicillin-streptomycin, 2 mm of L-glutamine, 1 g/l [U-^13^C] glucose, 1 g/l glucose (Sigma). One plate was treated with (2 μM) NEN while a second plate (control) was treated with dimethyl sulfoxide (DMSO) for 6 or 12 h. After these two incubation time points were performed, cell lysate was collected and the cells metabolites were obtained using a cold methanol- chloroform extraction process as previously described^49^. Briefly, cells were washed twice with cold phosphate buffer solution, and then trypsinized to collect the cell pellets. After that, the cell pellets were extracted with ice-cold methanol–chloroform-water (2-1-1) and centrifuged (18,000 g) for 15 min at 4 °C. Next, aqueous supernatants were collected and dried by using an evaporator. Samples were stored at −20 °C until they were used for NMR experiments.

### NMR analysis

Cell metabolites were redissolved in 600 µl of D_2_O containing 0.1% DSS, which used as an internal reference for proton and carbon NMR spectroscopy, and the samples were filter by using 0.2 µm pore size filter. NMR (2D) [^13^C-^1^H]-Heteronuclear Single Quantum Coherence  (HSQC) spectra were obtained on Bruker (Bruker Biospin, Karlsruhe, Germany) Avance II 800 MHz NMR spectrometer equipped with a 5-mm triple-resonance cryoprobe, thermostated to a sample temperature of 25 °C. 2048 complex points were acquired along the^1^H-dimension for each of the 64 complex points in aliphatic ^13^C dimension. The sweep width in proton and carbon dimensions were 13 and 120 ppm, respectively. The data were processed using NMRPipe software (www.nmrpipe.com) and then analyzed using Sparky 3.2 (University of California, San Francisco).

### Cell cycle profile

MC38 or HCT116 cells were treated with different concentrations of NEN or oxyclozanide for 24 or 48 h, whereas the control group was treated with DMSO. Cells were then fixed with ice-cold 70% ethanol on ice for 30 min. After that, 5 μl of PI (Sigma, P 4170) (1 mg/ml) solution and 50 μl of RNAse A (Sigma, R-4875) solutions were added to the fixed cells, which were then kept for 30 min in dark at room temperature prior analysis by flow cytometry.

### Cell viability assay

MC38 or HCT116 cells were plated in six well plates at 1 × 10^5^ cells/2 ml medium in each well and incubated overnight. After overnight culture, vehicle control or drugs were added at different concentrations. Each experiment was done in triplicate. After 24 h incubation with drug at 37 °C, cell viability was determined by staining with trypan blue and quantified by counting the cells in hemocytometer under normal light microscope.

### Clonogenicity assay

MC38 or HCT116 cells were plated in 6-well plates at 200 cells per well in 2 ml of DMEM. Cells were treated with NEN or oxyclozanide dissolved in DMSO with different concentrations for 24 or 48 h. After incubation, drugs were kept with medium for whole experiment period. Cells were maintained with changes of the medium plus drugs for 10 days. The colonies were then fixed in 1:3 Acetic–Methanol solution for 5 min, stained with 0.02% crystal violet for 20 min and counted under normal light microscopy.

### Cell invasion assay

Invasion assays were performed with a Boyden chamber (Corning, Corning, NY). Prior to the experiment, MC38 cells were plated at concentration of 2 × 10^6^ per 10 cm dish and incubated for 24 h. Cells were washed with PBS and then maintained with serum—free medium and 1μl NEN or DMSO for 4 h. Cells were trypsinized, and cell pellets were collected by centrifugation at (1500 × g) for 5 min. Cells were resuspended and 2 × 10^6^ cells (200 μl) were seeded into a transwell containing serum—free medium with 1μM NEN or vehicle alone. 700 μl complete medium supplemented with 1μl NEN or DMSO was added to the lower chambers. Plates were kept at 37 °C and 5% CO_2_ for 18 h. Afterwards, the inserts were removed, cells fixed with 70% cold methanol for 5 min, followed by incubation with 0.2% crystal blue for 10 min at the dark. Inserts were then rinsed twice with PBS and left to completely dry. Invaded cells were counted in 4 different fields by using Neubauer chambers slide. Each invasion experiment was performed in duplicate.

### Wound healing assay

MC38 or HCT116 cells were plated in six-well plates, growing to confluence. Wounds were made by using a 10 μl pipette tip to scratch through confluent cell monolayer. Fresh medium was added to the plates and the initial wound was recorded by taking images at (0 h) time. The first and second groups were treated with various concentrations of NEN or oxyclozanide at different time points whereas the control group was treated with DMSO. Images of each time point were recorded. Wound closure percentage was determined by comparing the wound closure size between the initial wound and the different time points in control vs. treated groups.

### In vivo intestinal polyps

Male APC^min/+^ mice (20) were purchased from The Jackson Laboratory (Farmington, CT). The mice were distributed randomly into two groups (10) per each group. The control group was fed with normal control diet (AIN-93M) while the treated group was fed with normal diet containing 1500ppm NEN for 8 weeks. After an 8-week treatment period with NEN the mice were sacrificed and the intestinal polyps were recognized and counted.

### Tumor xenograft experiments

Male NOD-scid-gamma (NSG, NOD.Cg-Prkdc^scid^ Il2rg^tm1Wjl^/SzJ) mice were ordered from The Jackson Laboratory (Farmington, CT). The mice were kept in vivarium of Rutgers -RWJMS. All experiments were done under an approved protocol (I12-069) by the Institutional Animal Care and Use Committees (IACUCs). Sub-confluent cultures of MC38 cells were harvested with trypsin-EDTA solution. Cells were counted and collected by centrifugation, and pellet was suspended in free-serum DMEM.

For intra-splenic injections, 30 male NSG mice were used. Mice were injected (0.1 mg/kg) buprenorphine subcutaneously for analgesia 30 min before the surgery. Then, the mice were injected anesthetic drugs i.p. (Acepromazine 5 mg/kg, and ketamin 100 mg/kg body weight). After anesthesia, the surgical site was cleaned and aseptically prepared by using iodophor and 70% alcohol. The spleen was pulled out the body of mice, the lower end of the spleen was circled with a 5/0 silk synthetic suture, and 1 × 10^5^ murine colon adenocarcinoma cells (MC38) in 0.2 ml medium were injected into the lower pole of the spleen and then we were waited for 10 min. Then, the spleen was ligated and the whole spleen was completely removed from the mouse body. The abdominal wall was closed with two layers of sutures. Postoperatively, mice were kept warm on a heating pad, which was disinfected with 70% ethanol in advance, and returned to their cages when completely awake. 10 h later, all mice received the second dose of (0.1 mg/kg) buprenorphine. Animals were assigned into three different groups: control group was fed with normal control diet (AIN-93M) (Research Diet, New Brunswick, NJ) while the first and second treated groups were fed with AIN- 93M containing 800 ppm oxyclozanide or with AIN-93M containing 2000 ppm NEN respectively. 3 weeks after the surgery, mice were sacrificed by cervical dislocation. Liver tissue was obtained and imaged. Tumor metastases were counted, tumor volume (in mm^3^) was calculated from recently excised liver by using an electronic caliper, using the next equation: [volume = 0.5 ^x^ (width)^2^ X length]^50^. Immediately a section of each normal tissue or tumor was fixed 10% neutral formalin for 24 h and then processed for histological and immunohistochemistry analysis, whereas the remaining liver tissue was kept in liquid nitrogen for Western blot or other analyses.

### Immunoblotting assay

MC38, HCT116 cells were plated onto six-well plate and maintained with DMEM to the 70% confluence. Cells were incubated with NEN or oxyclozanide at different concentrations for 2 h. The protein was extracted, separated through sodium dodecyl sulfate polyacrylamide gel electrophoresis, and moved to polyvinylidene difluoride membranes (Millipore, Billerica, MA) as previously described previously^34^. Immunoblotting was performed with primary antibody (1:1000 dilution) followed by secondary antibodies (Santa Cruz Biotechnology, Dallas, TX). Proteins were detected using enhanced chemilluminescent Western blot reagents (Amersham, 95038-566) (Amersham, Piscataway, NJ).

### Liver histology

Liver tissues were collected after the mice were euthanized. Samples were kept in 10% neutral buffered formalin for 24 h, changed to 70% ethanol alcohol and later implanted in paraffin. Liver tissue sections were completed and stained with H&E stain. Images were taken with a Carl Zeiss Universal Microscope imaging system with different phase-contrast objectives.

### Statistical analysis

Results are expressed as means ± SD experiments. Data were evaluated using student *t* test to compare the control group and drug treated group, statistical significance *P*-values were indicated as *, *P* < 0.05; **, *P* < 0.01; and ***, *P* < 0.001.

## Electronic supplementary material

CDDIS-17-0930-T-s02.docx

CDDIS-17-0930-T-s03.pdf

CDDIS-17-0930-T-s04.pdf

CDDIS-17-0930-T-s05.pdf

CDDIS-17-0930-T-s06.pdf

CDDIS-17-0930-T-s07.pdf

CDDIS-17-0930-T-s08.pdf

CDDIS-17-0930-T-s09.pdf

CDDIS-17-0930-T-s10.pdf
