# Supplementary material for: Effect of mitochondrial uncouplers niclosamide ethanolamine (NEN) and oxyclozanide on hepatic metastasis of colon cancer
Source: Cell Death Dis. 2018 Feb 13;9(2):215. doi: 10.1038/s41419-017-0092-6 (PMC5833462; doi:10.1038/s41419-017-0092-6)
Supplement: Supplementary file 1 — CDDIS-17-0930-T-s02.docx [file 41419_2017_92_MOESM1_ESM.docx]

**Supplementary Material:**

**Supplementary materials contain one supplementary table (Table S1) and 7 supplementary figures (Figures 1-7).**

**Table legend**

**Supplementary Table S1.** Identification of glucose metabolites based on 2D13 C-1H- HSQC spectrum. Colon cancer cells were fed [U-13C] glucose.

**Supplementary Figure S1.** Determination of the efficacious NEN and oxyclozanide concentrations that uncouple mitochondria in HCT-116 cells. NEN (**a**) or oxyclozanide (**b**) cells were treated with NEN or oxyclozanide at various concentrations and mitochondrial membrane potential was measured with TMRE staining, scale bars, 200 μm.

**Supplementary Figure S2.** Effect of the NEN and oxyclozanide on plasma membrane potential of MC38 cells using DiBAC_4_(3) dye staining. (a-b) Representative flow cytometry results of the effect of NEN and oxyclozanide on the plasma membrane potential of MC38 cells. (**c-d**) quantification of the effect of NEN and oxyclozanide on plasma membrane potential from three independent experiments. MC38 cells were treated with NEN or oxyclozanide at various concentrations and plasma membrane potential was measured with DiBAC_4_ (3) dye followed by flow cytometry analyses. For quantification, Mean intensity of DMSO treated cells was set as 100, the relative changes were normalized with DMSO treated cells. Increase in fluorescence intensity is indicative of reduction in plasma cell membrane potential. All results are represented means ± SD from triplicate experiments.

**Supplementary Figure S3.** Representative 2D 13C-1H- HSQC spectrum of colon cancer MC38 cells untreated (blue) or NEN treated (Magenta) acquired on a 800MHz NMR spectrometer at 25oC. The cross peaks analyzed in the data shown in the Figure 2 are labeled. Abbreviations: Lac, lactate; Glu, glutamate; Gln, glutamine; Gly, glycine; Ser, serine.

**Supplementary Figure S4.** NEN affects cell cycle progression and reduces colony formation of human colon cancer HCT 116 cells. (**a- c**) cell cycle profile of HCT116 cells treated with DMSO vehicle (a) or 2.0 μM NEN (b) for 24h, with percentage of cells in each phase summarized in (c). (d) Cell viability of HCT116 cells after a 24 h treatment with NEN at various concentrations, detected by trypan blue exclusion assay. (**e**) Clonogenicity of HCT116 cells, cells were grown in medium containing NEN at various concentrations, as indicated, for 10 days, and the colonies formed were counted. Results from **(d-e**) are shown as means ± SD from three independent experiments and statistical significance (P) between the control and treated cells was determined by student t-test. ***, *P* < 0.001.

**Supplementary Figure S5.** Effect of oxyclozanide on cell cycle progression and clonogenicity of MC38 cells. (**a**) oxyclozanide causes cell cycle arrest, MC38 were treated with (40 μM) oxyclozanide for 24h and percentage of cells at each phase was determined by flow cytometry assay. (**b**) oxyclozanide reduces cell viability, MC38 cells were treated with different concentrations oxyclozanide for 24 h, and then the cell viability was detected by trypan blue staining. Values expressed means ± SD from triplicate experiments. (**c**) The effect of oxyclozanide on colony formation in MC38 cells. All results are represented means ± SD from triplicate experiments and (*P*) value was detected by (ANOVA) (**P* < 0.05; ***P* < 0.01; ****P* < 0.001 vs. DMSO control).

**Supplementary Figure S6** NEN reduces colon cancer cell invasion and migration*.* (a-b) Boyden chamber assay showing representative pictures of stained cell migrated through the trans-wells, either under control condition (a), or in the presence of 1 μM NEN. Scale bars, 50 μm. (c) Quantification of (a-b). (d) Representative pictures showing wound closure of cells treated with vehicle (DMSO) or NEN (either 2 μM or 5 μM as indicated) at 0, 8, 12 h time points. (e) Quantification of percentage of wound closure, under each condition; the gaps were measured at 5 different positions and averaged. Results shown as means ± SD. Scale bars, 20 μm. Statistical significance (*P*) was determined by student-t test between the control and drug treated cells. **, P<0.01; ***, *P* < 0.001. All data show representative results from three independent experiments.

**Supplementary Figure S7** Oxyclozanide reduces colon cancer cell migration*.* (a) Representative pictures showing wound closure of MC38 cells treated with vehicle (DMSO) or oxyclozanide (either 20 μM or 40 μM as indicated) at 0, 8, 12 h time points, as indicated. (b) Quantification of percentage of wound closure. Under each condition, the gaps were measured at 5 different positions and averaged. Scale bars, 20 μm. Results shown as means ± SD. Statistical significance (*P*) was determined by student-t test between the control and drug treated cells. **, P<0.01; ***, *P* < 0.001. All data show representative results from three independent experiments.
