# Supplementary material for: Effect of mitochondrial uncouplers niclosamide ethanolamine (NEN) and oxyclozanide on hepatic metastasis of colon cancer
Source: Cell Death Dis. 2018 Feb 13;9(2):215. doi: 10.1038/s41419-017-0092-6 (PMC5833462; doi:10.1038/s41419-017-0092-6)
Supplement: Supplementary file 2 — CDDIS-17-0930-T-s03.pdf [file 41419_2017_92_MOESM2_ESM.pdf]

Supplementary Table1. 2D ^{13}C-^{1}H-HSQC spectrum of colon cancer cell were acquired based on [U-^{13}C]glucose labeling.

Number	Metabolites	Carbon position	^{1}H chemical shift (ppm)	^{13}C chemical shift (ppm)
	Amino acids			
1	Glutamate	Cβ	2.07, 2.13	29.58
	Glutamate	Cγ	2.36	36.22
2	Glutamine	Cγ	2.46	33.56
3	Glycine	Cα	3.57	44.20
4	Serine	Cβ	3.58	57.26
	Organic acids			
5	Lactate	Cα	4.12	71.22
	Nucleotides			
6	UTP / UDP	C2	4.39	76.49
