# Supplementary figures and images for: Effect of mitochondrial uncouplers niclosamide ethanolamine (NEN) and oxyclozanide on hepatic metastasis of colon cancer
Source: Cell Death Dis. 2018 Feb 13;9(2):215. doi: 10.1038/s41419-017-0092-6 (PMC5833462; doi:10.1038/s41419-017-0092-6)

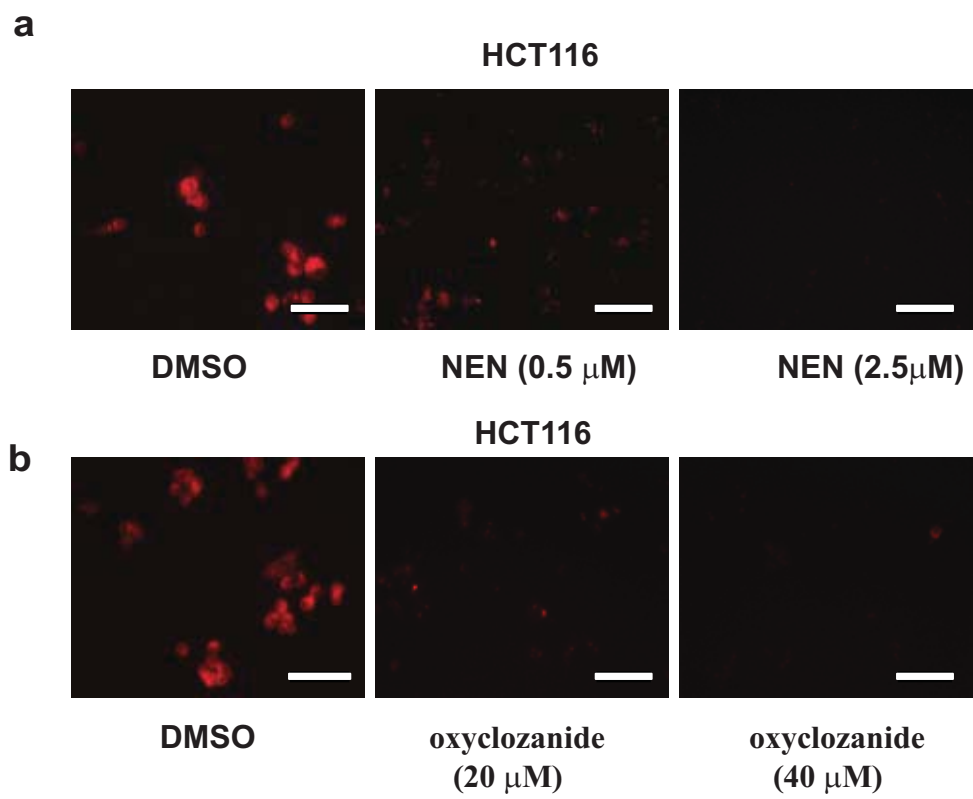

**Figure S1**

Supplement: Supplementary file 3 — CDDIS-17-0930-T-s04.pdf [file 41419_2017_92_MOESM3_ESM.pdf]

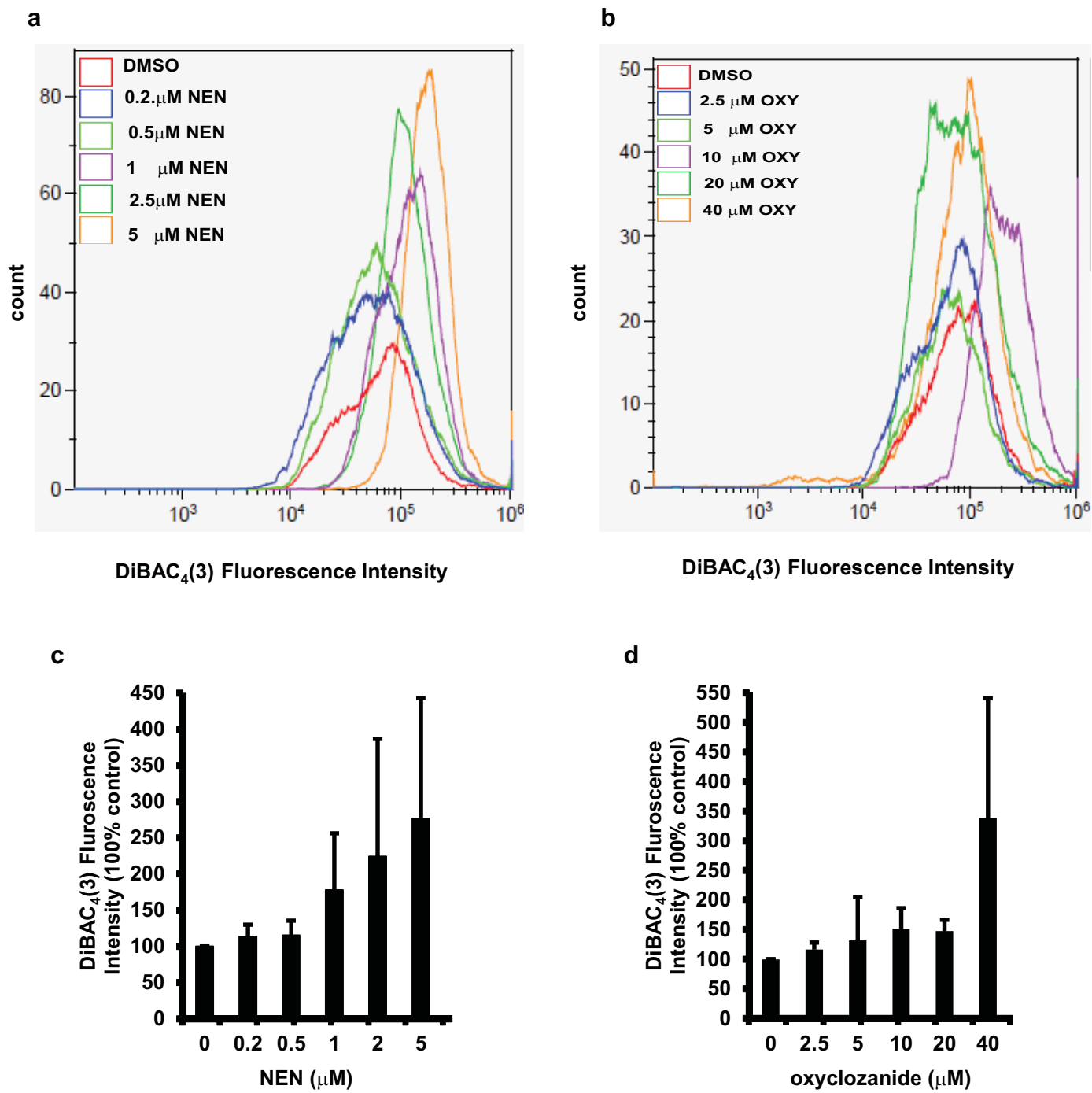

Figure S2

Supplement: Supplementary file 4 — CDDIS-17-0930-T-s05.pdf [file 41419_2017_92_MOESM4_ESM.pdf]

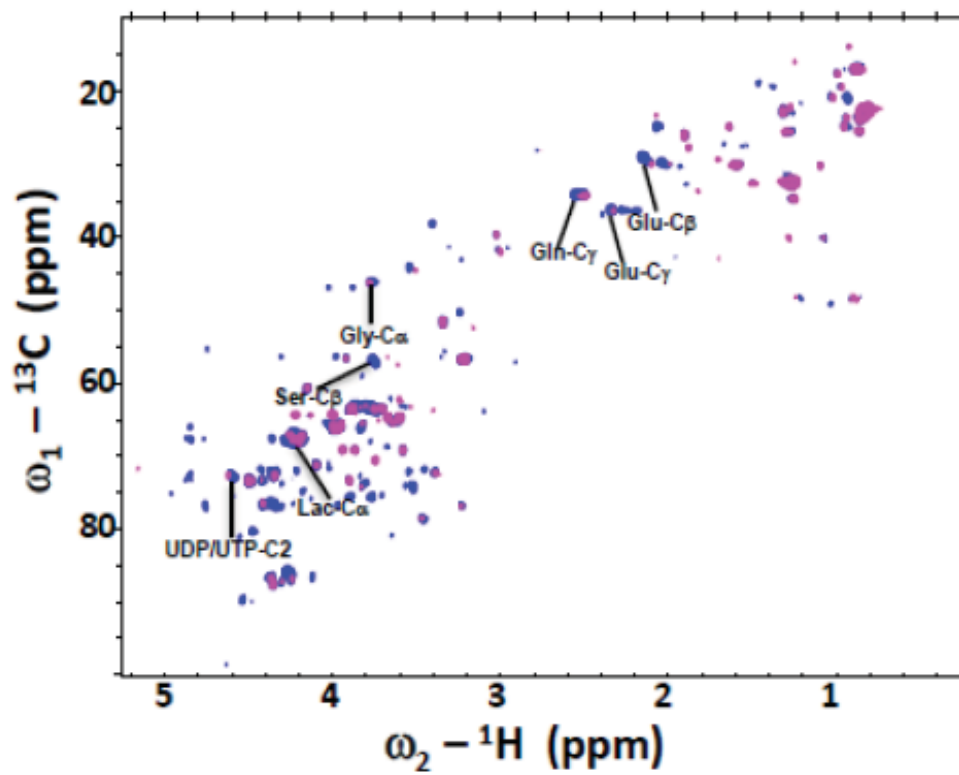

Figure S3

Supplement: Supplementary file 5 — CDDIS-17-0930-T-s06.pdf [file 41419_2017_92_MOESM5_ESM.pdf]

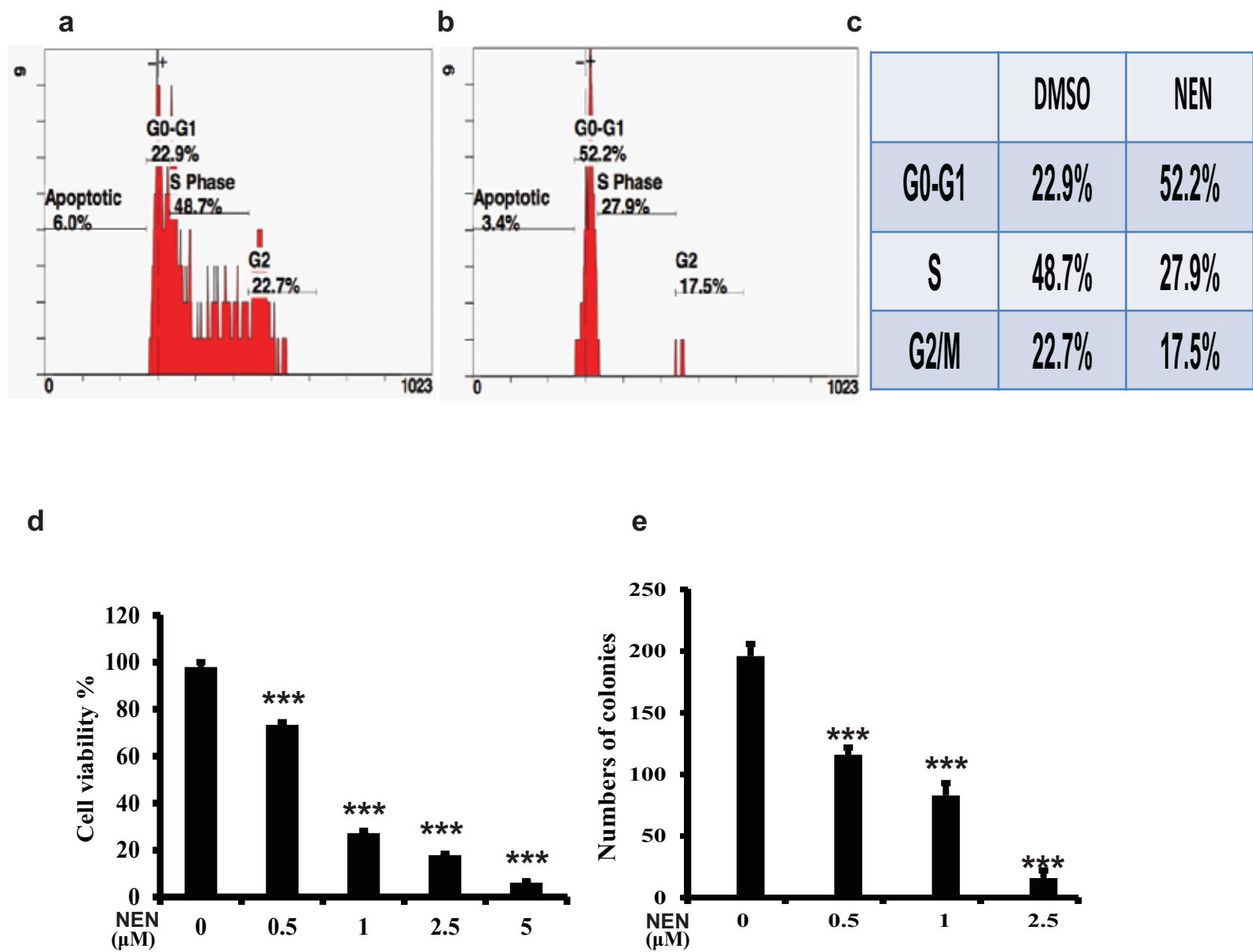

Figure S4

Supplement: Supplementary file 6 — CDDIS-17-0930-T-s07.pdf [file 41419_2017_92_MOESM6_ESM.pdf]

**a**

|       | DMSO   | Oxyclozanide |
|-------|--------|--------------|
| G0-G1 | 30.20% | 46.04%       |
| S     | 47.84% | 35.18%       |
| G2/M  | 19.30% | 5.45%        |

**b**

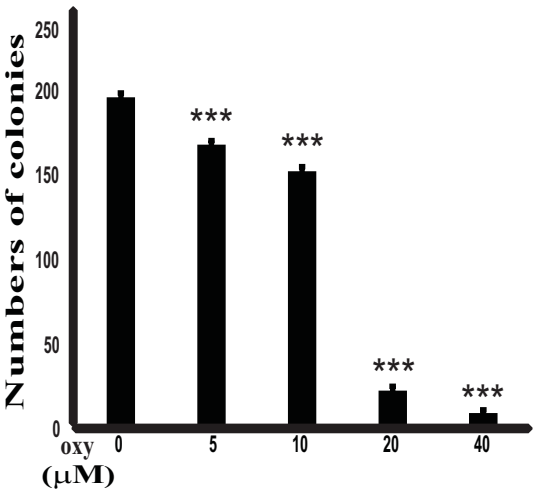

**c**

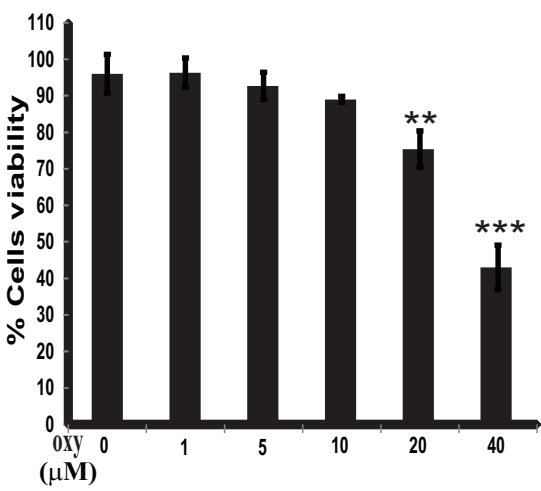

**Figure S5**

Supplement: Supplementary file 7 — CDDIS-17-0930-T-s08.pdf [file 41419_2017_92_MOESM7_ESM.pdf]

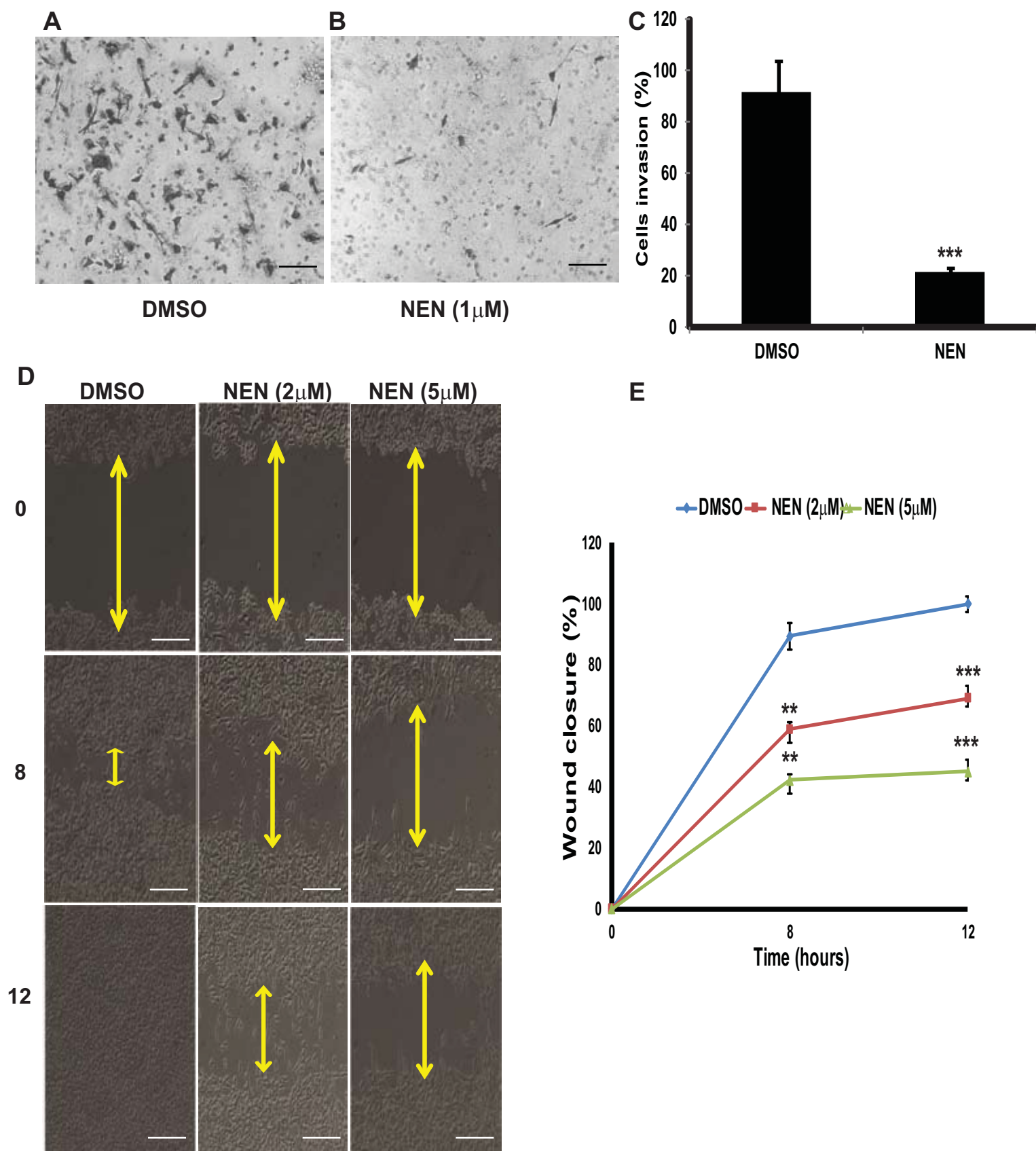

Figure S6

Supplement: Supplementary file 8 — CDDIS-17-0930-T-s09.pdf [file 41419_2017_92_MOESM8_ESM.pdf]

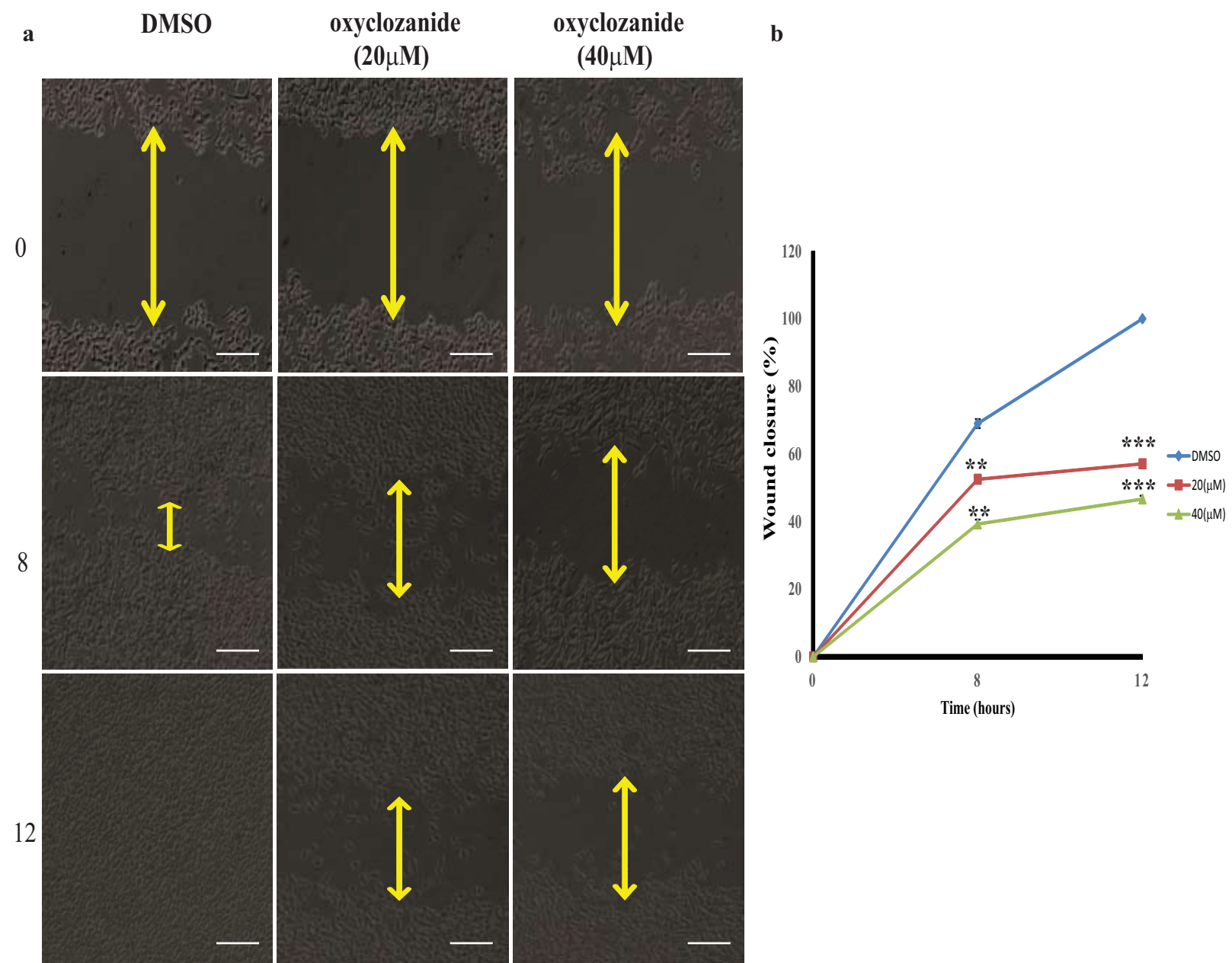

**Figure S7**

Supplement: Supplementary file 9 — CDDIS-17-0930-T-s10.pdf [file 41419_2017_92_MOESM9_ESM.pdf]
